# Quinoa husk peptides reduce melanin content via Akt signaling and apoptosis pathways

**DOI:** 10.1016/j.isci.2022.105721

**Published:** 2022-12-05

**Authors:** Caijing Han, Bingjie Lin, Xinyu Huang, Zhaojie Mao, Xiaoting Kong, Lei Fang, Peng Xue, Anning Wang, Fengxiang Zhang

**Affiliations:** 1 School of Public Health, Weifang Medical University, Weifang, 261053 Shandong, China; 2 Neurology Department, The First Affiliated Hospital of Weifang Medical University (Weifang People’s Hospital), Weifang, 261000 Shandong, China

**Keywords:** Medical biochemistry, plant biochemistry, cellular physiology

## Abstract

To improve the treatment of pigmentation disorders, looking for natural and safe inhibitors of melanin synthesis has become an area of research interest. The quinoa husk peptides reportedly elicit various biological activities (e.g., anti-cancer, antioxidant, anti-hypertensive, and so forth), but its effects on melanin inhibition remain unknown. In the current study, we purified quinoa husk peptides with 30 and 80% ethanol using a macroporous adsorption resin (DA201-C). Component screening revealed that the 80%-ethanol fraction (i.e., QHP fraction) contained numerous short peptides (84.41%) and hydrophobic amino acids (45.60%), while eliciting a superior tyrosinase [TYR]-inhibition rate, 2,2-diphenyl-1-picryhydrazil-scavenging rate, reducing activity, and chelating capacity compared to the 30% fraction and was thus applied in subsequent analyses. Differentially expressed genes in the QHP fraction were primarily enriched in the Akt-signaling pathways based on transcriptomics. Thus, we assessed the expression of related proteins and genes in A375 cells and rat skin cells following treatment with QHP.

## Highlights

• The melanin inhibition effects of Quinoa husk peptides are unknown yet
• Akt-signaling pathways were further researched on the base of transcriptomics
• Quinoa husk peptides reduce melanin content through Akt signaling and apoptosis

## Graphical abstract

**Figure:**
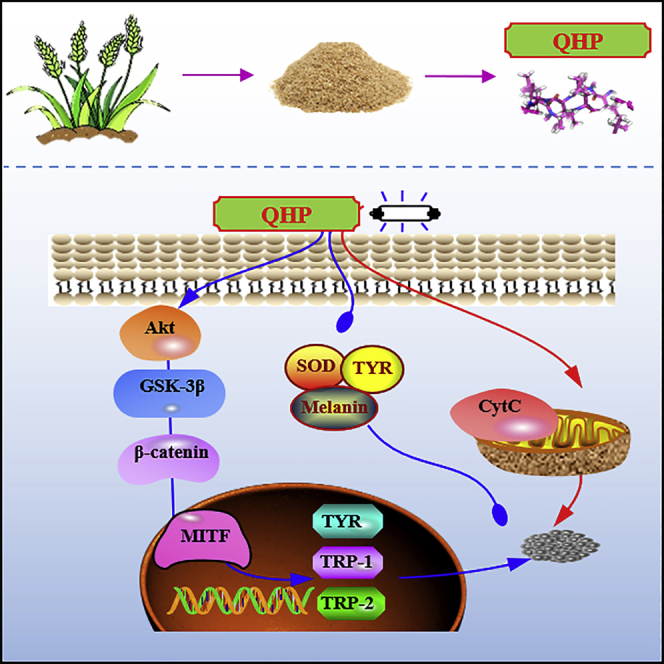

## Introduction

Melanin is essential for protecting human skin from radiation; however, abnormal melanin accumulation contributes to skin aesthetic issues, which can lead to the formation of freckles, black spots, chloasma, and bask-in-spot, thus affecting people’s psychological state and quality of life.^1^ Recently, many compounds, including hydroquinone, arbutin, kojic acid, glucocorticoids, and mercuric chloride, have been applied for the treatment of pigmentation disorders; however, their application is limited by their low stability, poor skin permeability, low activity, and cytotoxicity, as well as their induction of dermatitis and erythema following long-term use.^2^^,^^3^^,^^4^ Hence, research has begun to focus on screening effective natural compounds for the treatment of various dermatologic conditions.^5^^,^^6^^,^^7^ In particular, foodborne inhibitors have received considerable attention. For instance, peptide CT-2 (Leu−Gln−Pro−Ser−His−Tyr) from rice bran protein can potently inhibit melanogenesis in mouse B16 melanoma cells, suggesting its potential for treating melanin-related skin disorders.^8^ Moreover, a novel peptide (Met-Gly-Arg-Tyr) isolated from marine microalgae attenuates oxidative stress and melanogenesis in B16F10 melanoma cells.^9^

Quinoa contains high levels of proteins, minerals, vitamins, and phytochemicals, whereas its husks—generated through food product processing—contain approximately 16% protein.^10^^,^^11^ The quinoa husks—accounting for 8-12% of quinoa processing waste—are typically burned or discarded,^12^ leading to a waste of quinoa protein. However, protein hydrolyzed peptides of quinoa have a variety of biological activities, including antioxidant, anti-diabetic, anti-cancer, anti-hypertensive, and anti-inflammatory activities.^13^^,^^14^^,^^15^^,^^16^^,^^17^ Nevertheless, the melanin-inhibiting effects of quinoa polypeptides have not yet been investigated. Therefore, the current study sought to take advantage of quinoa waste (quinoa husks) to extract protein to assess their effects on melanogenesis.

**Figure 1:**
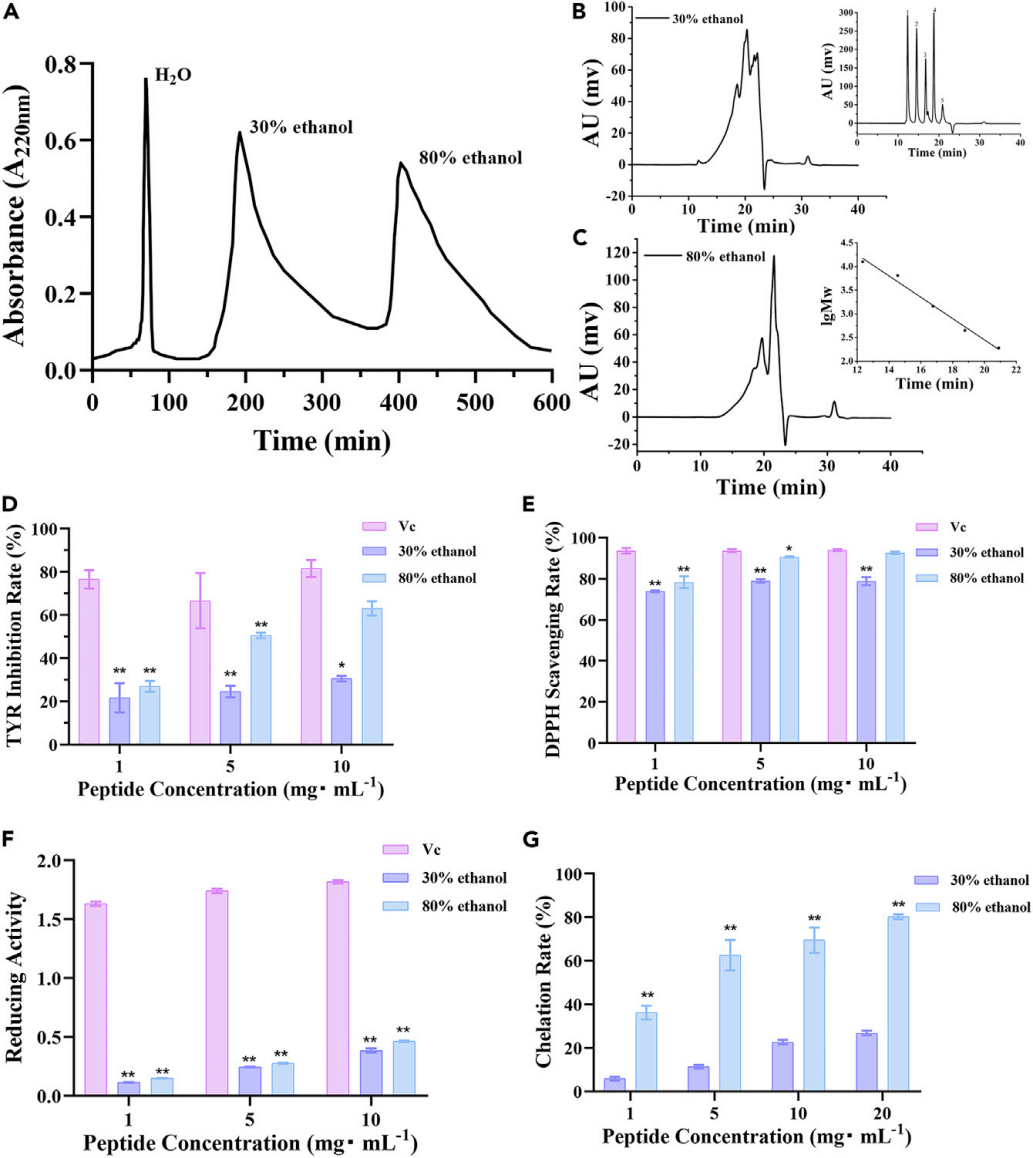
Purification and screening of quinoa husk peptides (QHP) (A) The purification of QHP with different concentrations of ethanol. (B) Molecular weight of eluting components with 30% ethanol. (C) Molecular weight of eluting components with 80% ethanol. (D–G) The screening of quinoa husk peptides of TYR inhibition (D) DPPH scavenging rate (E) Reducing activity (F) Chelation rate (G) All experiments were performed in triplicate, n = 3; ∗p < 0.05 versus the Vc group (positive group). ∗∗p < 0.01 versus the Vc group (positive group).

More specifically, we extracted proteins from quinoa husk, hydrolyzed them into polypeptides using alkaline protease, and subsequently gradient purified them with 30 and 80% ethanol using a macroporous adsorption resin (DA201-C). The components in the 80%-ethanol fraction (designated the quinoa husk polypeptide [QHP] fraction) exhibited superior tyrosinase (TYR)-inhibitory activity and antioxidant activity (Figure 1). Therefore, we aimed to further demonstrate (I) the impact and mechanism of QHP on melanin synthesis in A375 melanoma cells, and (II) the influence and mechanism of UV B (UVB)-induced hyperpigmentation in rat skin. Our specific aims were as follows: (I) purify quinoa husk peptides with 30 and 80% ethanol and screen the optimum active components; (II) examine the effects of QHP on cell viability, TYR activity, and melanin content; and (III) investigate the potential underlying mechanisms by performing transcriptome sequencing, western blot analysis, quantitative reverse transcription-polymerase chain reaction (qRT-PCR), and apoptosis assays. The purpose of this article was to research the mechanism of reducing melanin content in quinoa husk peptides.

## Results and analysis

### Purification and screening of C-quinoa husk polypeptide

The peptides (C-QHP) were gradient-purified using 30 and 80% ethanol after binding to the macroporous adsorption resin, DA201-C. Two fractions, labeled the 30 and 80% ethanol eluates, were collected and freeze-dried (Figure 1A), and amino acid compositions (Table 1) and their molecular weights (Figure 1B and Table 2) were determined. Both eluates primarily comprised small molecular weight peptides (<3000 Da), accounting for 84.41 and 81.41% of each sample, respectively. In addition, the proportion of hydrophobic amino acids of the 80%-ethanol fraction (45.60%) was higher than that of the 30%-ethanol fraction (39.76%). Moreover, the TYR-inhibition rate, DPPH-scavenging rate, reducing power, and chelation rate were markedly higher in the 80% fraction (Figures 1D–1G). In fact, no significant differences were noted in these activities between the 80% fraction and positive-control group (Vc group) at 10 mg/mL, particularly in terms of the TYR-inhibition and DPPH-scavenging rates. Therefore, we selected the 80%-ethanol fraction (QHP fraction), the peptide content of which was approximately 87.64%, for subsequent testing.

**Table 1:** Amino acid composition (per 1000)

Amino Acid Species	Amino Acid	30% Ethanol Component	80% Ethanol Component
Hydrophobic amino acid	Val	69	72
	Ile	50	62
	Leu	82	100
	Phe	41	61
	Ala	81	69
	Met	20	14
	Pro	55	78
Hydrophilic amino acid	Thr	55	46
	Ser	67	55
	Lys	33	35
	His	13	18
	Asp	122	90
	Glu	142	102
	Gly	86	95
	Cys	16	19
	Tyr	34	40
	Arg	35	44
Hydrophobic amino acid/total amino acid (%)		39.76%	45.60%

**Table 2:** Molecular weight (Da) ratio (%)

Component Types	Molecular weight(Da)	Ratio (%)
30% Ethanol Component	＞3000	5.63
	100–3000	81.41
	＜100	12.96
80% Ethanol Component	＞3000	4.39
	100–3000	84.41
	＜100	11.20

### Dose screening and effect of quinoa husk polypeptide on A375 melanoma cells

**Figure 2:**
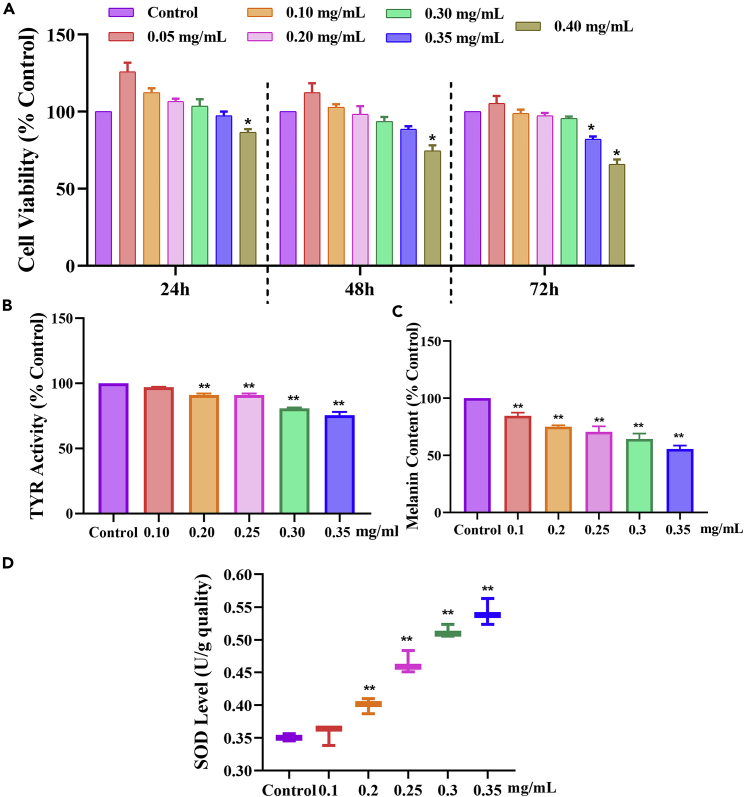
Dose screening and effect of QHP on A375 melanoma cells (A) Cell viability of culturing 24, 48, and 72 h. (B) TYR activity of culturing at 48 and 72 h. (C) Melanin content of culturing 48 and 72 h. (D) Effect of QHP on SOD in A375 cell; The control group did not undergo any treatment, namely the sample concentration was 0 mg/mL; Cell viability was performed 6 times, other experiments were performed in triplicate, n = 3; ∗p < 0.05 versus the control group. ∗∗p < 0.01 versus the control group.

The effect of QHP on cell viability was assessed by performing CCK-8 cell-proliferation assays at different QHP concentrations (0.10-0.40 mg/mL) for different times (24-72 h). Cell viability decreased after exposure to 0.35 mg/mL QHP for 72 h or 0.4 mg/mL for 24-72 h, when compared with those of the control group (p < 0.05), demonstrating that QHP were toxic to cells (Figure 2A). Considering that excessively long incubation times can lead to cell senescence, the experiment was carried out at concentrations of 0.1-0.35 mg/mL for 48 h. With increased QHP concentration, TYR activity gradually decreased (Figure 2B) and differed significantly from that of the control group at concentrations of 0.2-0.35 mg/mL (p < 0.01). The same trend was observed for melanin content with significant differences observed at concentrations of 0.10-0.35 mg/mL (p < 0.01; Figure 2C). SOD is an important free radical scavenging enzyme, the activity of which is essential for regulating free radicals. QHP enhanced SOD activity in a dose-dependent manner (Figure 2D), suggesting that it may enhance free radical scavenging, thus, exhibiting antioxidant activity.

### Effect of quinoa husk polypeptide on apoptosis

**Figure 3:**
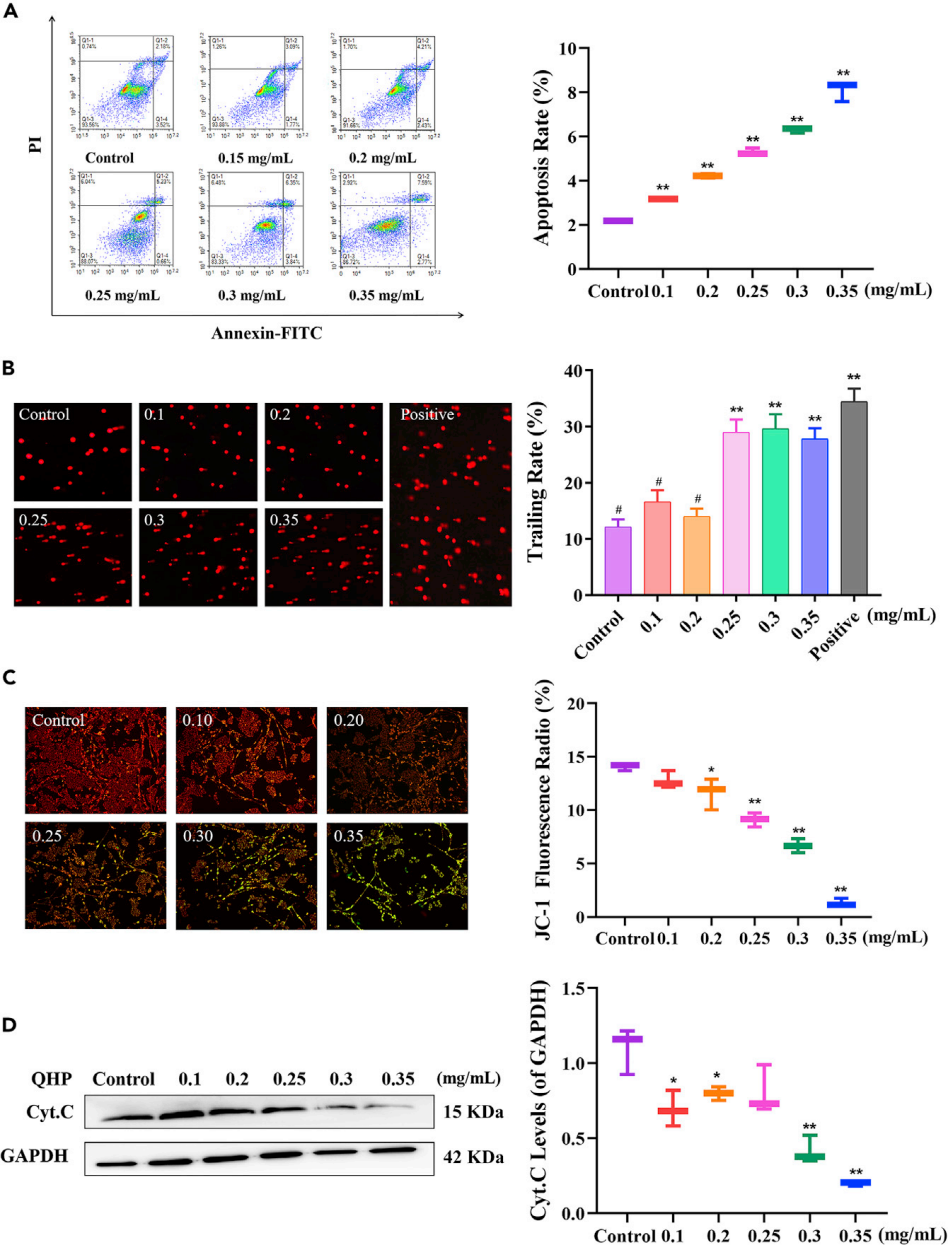
Effect of QHP on apoptosis (A) QHP accelerated the A375 cells apoptosis by flow cytometric analysis stained with Annexin V-FITC and PI. (B) QHP accelerated DNA-damage of A375 cells by comet assay. (C) QHP accelerated mitochondrial apoptosis in A375 cells detected by Mitochondrial membrane potential. (D) Expression of mitochondrial apoptosis protein cytochrome *c* by western blotting. The control group did not undergo any treatment, namely the sample concentration was 0 mg/mL; All experiments were performed in triplicate, n = 3; ∗p < 0.05 versus the control group. ∗∗p < 0.01 versus the control group. #p < 0.05 versus the positive group.

A375 cell apoptosis was measured at the cell membrane level by flow cytometric analysis after staining with Annexin V-FITC and propidium iodide (PI). The rate of apoptosis gradually increased with increasing QHP concentrations, reaching 8.11% at a QHP concentration of 0.35 mg/mL (Figure 3). Moreover, significant differences (p < 0.01) were observed between the group treated with 0.1-0.35 mg QHP/mL and the control group, indicating that QHP promoted A375 cell apoptosis.

DNA damage in A375 cells was assessed with comet assays (Figure 3B). Undamaged DNA appears round without trailing, while damaged DNA exhibits trailing, similar to the shape of a comet. Compared with that of the control group, the DNA of which was round without trailing, the DNA of the QHP group exhibited obvious tailing at QHP concentrations of 0.25-0.35 mg/mL (p < 0.01), indicating that QHP treatment accelerated DNA damage. However, DNA damage also appeared in the positive-control group (cell stimulated with 150 μm H_2_O_2_) with no significant differences observed between QHP concentrations of 0.25-0.35 mg/mL and the positive control group. Hence, QHP elicited the same effect as the positive control group.

Many important apoptotic events are closely related to mitochondria, including the release of caspase activators, such as cytochrome *c* (Cyt C), changes in the electron-transport chain, loss of the mitochondrial membrane potential (ΔΨm), changes in the intracellular redox state, and the participation of Bcl-2 family members in promoting and inhibiting apoptosis. The transmission of different signals finally concentrates on the mitochondria to initiate or inhibit these events and their effects. Therefore, we measured ΔΨm values and Cyt C levels to determine whether A375 cell apoptosis was related to mitochondria. As shown in Figure 3B, QHP concentration was inversely proportional to the ΔΨm. With increasing QHP concentrations, the ΔΨm was destroyed (i.e., the JC-1 fluorescence changed from red to green), resulting in a depressed JC-1 fluorescence ratio. At concentrations of 0.25, 0.30, and 0.35 mg/mL, QHP significantly reduced the JC-1 fluorescence ratio to 9.10%, 6.66%, and 1.31%, respectively, compared to that of the control group (p < 0.01).

Cyt C is released from the inner mitochondrial membrane to the cytoplasm when stimulated by apoptotic signals. It then combines with apoptosis activator 1 (Apaf-1) to form an apoptotic complex, which triggers the caspase cascade to induce apoptosis. As shown in Figure 3C, the mitochondrial level of Cyt C gradually decreased as the QHP concentration increased, indicating that Cyt C was released into the cytoplasm during apoptosis. In particular, at concentrations of 0.30 and 0.35 mg/mL, QHP significantly reduced Cyt C expression to 0.42 and 0.20 that of GAPDH, respectively (p < 0.01). Taken together, these results showed that QHP accelerated A375 cell apoptosis at the cell membrane, DNA, and mitochondrial levels.

### Analysis of genes differentially expressed due to quinoa husk polypeptide treatment

**Figure 4:**
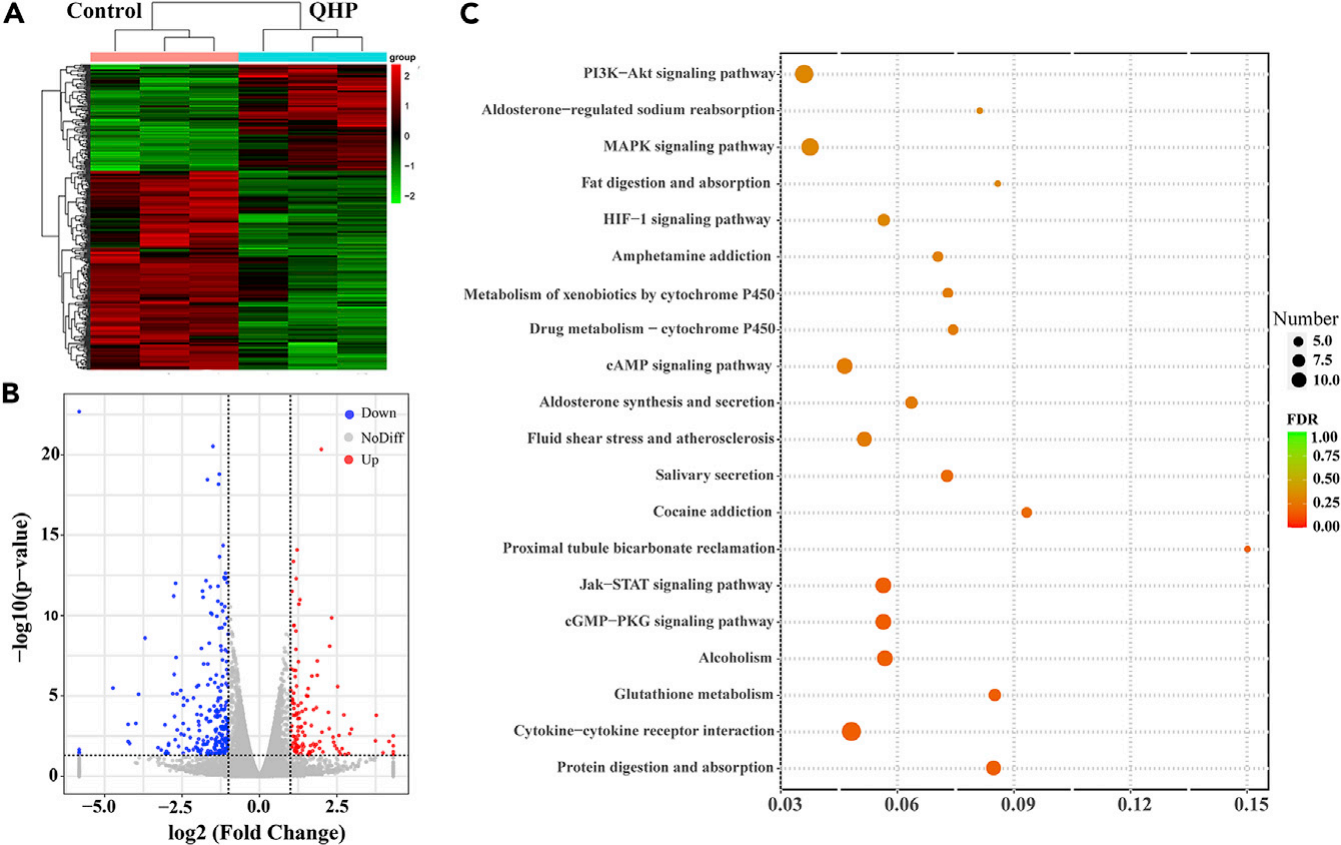
Analysis of genes differentially expressed due to QHP treatment (A) Clustering of differentially expressed genes; Horizontal indicates genes, and each column indicates a sample. Red indicates high-expression genes and green indicates low-expression genes. (B) Volcano map of differentially expressed genes; The two vertical dotted lines in the figure represent the threshold of 2-fold expression difference. The horizontal dotted line indicates the p value = 0.05 threshold. The red dots represent the up-regulated genes, the blue dots represent the down-regulated genes, and the gray dots represent the no significant differentially expressed genes. (C) KEGG enrichment analysis; According to the KEGG enrichment results, the enrichment degree is measured by rich factor, FDR value, and the number of genes enriched on this pathway. Where rich factor refers to the ratio of the number of differential genes enriched in the pathway to the number of genes annotated. The larger the rich factor, the greater the degree of enrichment. The general value range of FDR is 0-1. The closer it is to zero, the more significant the enrichment is. The top 20 KEGG pathways with the lowest FDR value, i.e. the most significant enrichment, are selected for display.

Although we have shown that QHP can inhibit TYR activity and reduce melanin content, the mechanism of action is unclear. Expression changes in key genes caused by QHP are also unknown; therefore, we first detected mRNA-expression differences via transcriptomics. Cluster analysis of the QHP and control groups revealed distinct sets of differentially expressed genes (Figure 4A). Moreover, the volcano plot indicates that 121 genes were up-regulated, while 226 were down-regulated in the QHP group compared to those in the control group (Figure 4B). Next, we used the Kyoto Encyclopedia of Genes and Genomes (KEGG) database to identify the 20 most enriched pathways (smallest p values) associated with the differentially expressed genes with the smallest p values (Figure 4C). Based on these findings, we selected the Akt-signaling pathway for subsequent analysis.

### Effect of quinoa husk polypeptide treatment on Akt-signaling pathway-related protein and mRNA expression levels

**Figure 5:**
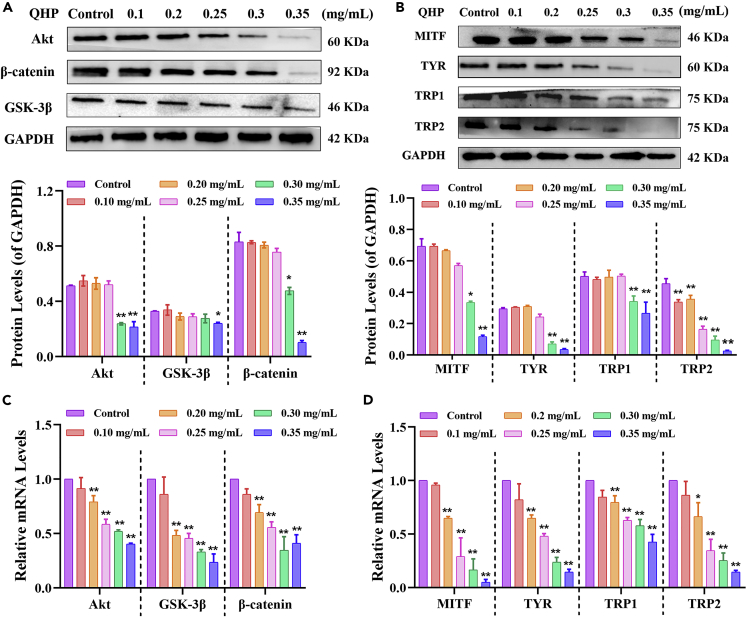
The effects of QHP on the expression and mRNA levels of related proteins in A375 cells (A–D) Different concentrations (0.1-0.35 mg/mL) of QHP acted on A375 cells for 48 h; The results of western blotting were relatively quantified: (A) The expression levels of Akt, GSK-3β, β-catenin; (B) The expression levels of MITF, TYR, TRP1 and TRP2; The results of RT-PCR were relatively quantified and all results were compared with the control group: (C) The mRNA levels of Akt, GSK-3β, β-catenin; (D) The mRNA levels of MITF, TYR, TRP1, and TRP2; The control group did not undergo any treatment, namely the sample concentration was 0 mg/mL; All experiments were performed in triplicate, n = 3; ∗p < 0.05 versus the control group. ∗∗p < 0.01 versus the control group.

MITF is the most important transcription factor related to melanin synthesis, and its products are largely involved in regulating the transcription of TYR, TRP-1, and TRP-2.^18^ Our results revealed that QHP regulated MITF via the Akt-signaling pathway (Figure 5). More specifically, QHP was found to inhibit the expression of Akt, GSK3β, and β-catenin, suggesting that QHP inhibited melanin synthesis through the Akt-signaling pathway. The expression levels of Akt, GSK3β, and β-catenin in the control group were 0.51, 0.33, and 0.83 that of GAPDH, respectively; these levels significantly decreased (p < 0.01 for each) by 0.21, 0.24, and 0.10 following treatment with 0.35 mg/mL QHP (Figure 5A). Down-regulation of β-catenin expression further regulated the expression of the nuclear protein MITF, which is involved in regulating the transcription of TYR, TRP-1, and TRP-2 (Figure 5B).

Subsequently, the mRNA expression levels were detected and those of Akt, GSK3β, and β-catenin were significantly decreased compared to those in the control group following treatment with 0.20-0.35 mg/mL QHP (p < 0.01 for each; Figure 5C). That is, the expression levels of MITF, TYR, TRP-1, and TRP-2 in the control group were 0.69, 0.30, 0.50, and 0.45 that of GAPDH, respectively, which significantly decreased (p < 0.01 for each) to 0.12, 0.04, 0.27, and 0.03, respectively, after treatment with 0.35 mg/mL QHP. In addition, their expression levels were lower than those in the control group after treatment following treatment with 0.30 mg/mL QHP (p < 0.05 for each). In particular, TRP-2 expression decreased significantly at experimental QHP concentrations ranging from 0.10 to 0.35 mg/mL (p < 0.01). Similarly, mRNA expression levels were measured (Figure 5D) and those of MITF, TYR, and TRP1 were significantly lower than those in the control group after incubation with 0.20-0.35 mg/mL QHP (p < 0.01), whereas the expression of TRP2 mRNA was significantly decreased with 0.25-0.35 mg/mL QHP (p < 0.01).

### Protective effects and the associated mechanism of quinoa husk polypeptide against UVB-induced injury in rats

**Figure 6:**
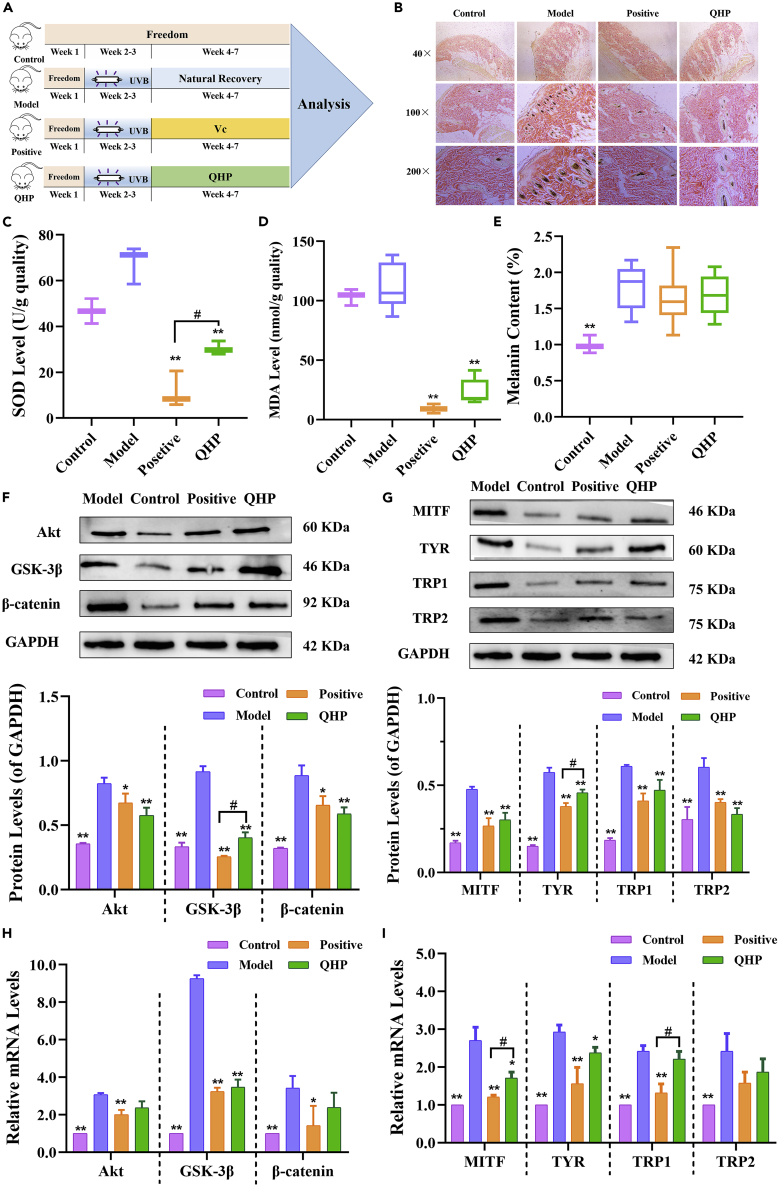
Protective effect and mechanism research of QHP on UVB-induced injury in rats (A) Schematic of the experimental design to examine the protection of QHP on the rat skin induced by UV induced injury. (B) The melanin content changes were shown by HE-stained skin in rats treated with the QHP for 28 days after UVB-induced damage. (C) Effect of QHP on SOD in animal skin. (D) Effect of QHP on MDA in animal skin. (E) Effect of QHP on melanin content in animal skin. (F) The results of western blotting were relatively quantified: F, The expression levels of Akt, GSK-3β, β-catenin. (G) The expression levels of MITF, TYR, TRP1, and TRP2. (H) The results of RT-PCR were relatively quantified: H, The mRNA levels of Akt, GSK-3β, β-catenin. (I) The mRNA levels of MITF, TYR, TRP1, and TRP2; The model group was treated with UVB-induced injury for two weeks. The positive group was treated with Vc for four weeks. The results are presented as the mean ± SD and they are representative of at least three independent experiments. ∗p < 0.05 versus the model group; ∗∗p < 0.01 versus the model group; #p < 0.05 versus the positive group.

The experimental scheme used to study the effect of QHP on UVB-induced rat skin injury is illustrated in Figure 6A. Hematoxylin and eosin (HE)-stained rat skin samples showed clear changes in melanin content among the different groups (Figure 6B). The positive group was significantly lower than the model group; The effect of the QHP group was not as good as that of the positive group, but it was also significantly lower than that of model group. We also measured the melanin content of rat skin, which confirmed the HE-staining results Figure 6E). The melanin content of QHP and positive groups was lower than that of the model group, and there was no difference between them (p > 0.05). Furthermore, the SOD and MDA levels were measured, which was very important to evaluate the antioxidant level. The SOD level of the QHP group was significantly lower than those in the model group (p < 0.01), but the effect was not as good as the positive group (p < 0.05) (Figure 6C). The MDA level of the QHP group was also significantly lower than those in the model group (p < 0.01), but the effect was close to the positive group (p > 0.05) (Figure 6D). These preliminary results indicate that melanin was related to antioxidation, consistent with previous findings.^2^

To explore the mechanism whereby QHP protects animal skin from UV radiation, the protein and mRNA expression levels of genes related to the Akt-signaling pathway were determined by western blotting and qRT-PCR. QHP treatment significantly inhibited the expression levels of Akt, GSK3β, and β-catenin compared with those in the model group (p < 0.01; Figure 6F), suggesting that QHP inhibited melanin through the AKT-signaling pathway. The inhibitory effects of QHP on Akt and β-catenin expression were even greater than those in the positive group. The mRNA-expression levels of them in the QHP group were lower than those in the model group, while that of GSK3β mRNA was significantly lower than that in the model group (p < 0.01; Figure 6H).

Next, we measured the expression of MITF and the downstream TYR, TRP-1, and TRP-2 at the protein and mRNA levels. Notably, the QHP group had significantly reduced the protein level of MITF, TYR, TRP-1, and TRP-2 compared to the model group (p < 0.01 for each), particularly the inhibition of TRP 2 was even better than the positive group (Figure 6G). Similarly, the mRNA expression was lower in the QHP group than in the model group, with that of MITF and TYR significantly lower in the QHP group than in the model group (p < 0.05 for both; Figure 6I).

**Figure 7:**
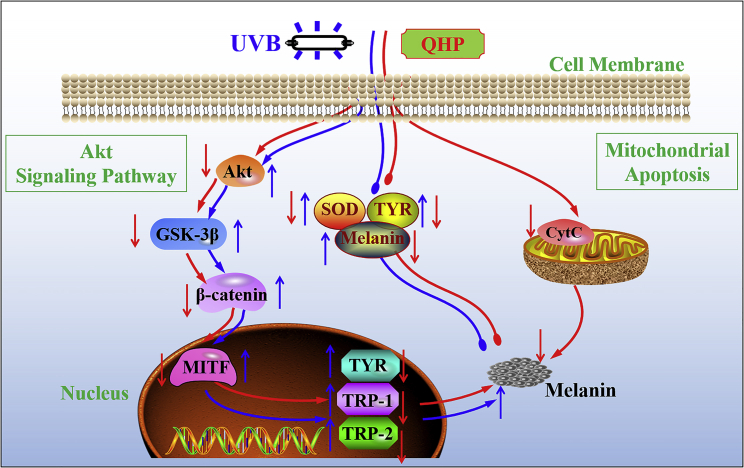
Schematic of the protective effects of QHP against UVB-induced injury skin The blue flow indicates the model group (UVB-induced injury), and the red flow indicates QHP group.

As depicted in Figure 7, our results revealed that QHP regulated MITF via the Akt-signaling pathway, which further regulated the expression of the nuclear proteins TYR, TRP-1, and TRP-2. Their expression levels were higher in the animal model group, resulting in increased melanin content. However, QHP treatment reduced the melanin content by down-regulating the abundance of proteins related to the Akt-signaling pathway. In addition, QHP treatment accelerated apoptosis and decreased the melanin content.

## Discussion

QHP activities are closely related to the amino acid composition and molecular weight^19^ In this study, we obtained two fractions (30 and 80% ethanol fractions) with the 80%-ethanol fraction (QHP) exhibiting higher activity. The molecular weight distribution and amino acid composition of both fractions were determined to investigate the cause of QHP high activities. The ratio of hydrophobic amino acids in QHP was 45.60%, whereas that in the 30%-ethanol fraction was 39.76%. More specifically, the Val, Ile, Leu, Phe, and Pro contents were 4.35%, 24%, 21.95%, 48.78%, and 41.82% higher, respectively, in QHP. TYR-inhibitory peptides preferentially contain Arg and/or Phe in combination with Val, Ala, and/or Leu.^20^ Indeed, Akihito Ochiai reported that peptide CT-2 (LEPSHY) from rice bran protein, which contained Leu and Pro, potently inhibited melanogenesis in mouse B16 melanoma cells.^8^ Meanwhile, Zhang et al. found that the rice proteolytic peptides LLK and LPK, which also contained Leu and Pro, decreased TRP-1 and TRP-2 expression, and affected melanin synthesis.^21^ Hence, we inferred that the average molecular weight of the QHP decreased while the hydrophobic amino acid content increased, particularly for Val, Ile, Leu, Phe, and Pro, which greatly influenced QHP activity, thus laying a foundation for our subsequent screening for peptides with defined sequences.

Apoptosis is a type of cell death that enables better adaptation to the living environment. Mitochondria play a considerable role in apoptosis, with diminished mitochondrial membrane permeability considered one of the earliest events of apoptosis.^22^ Subsequently, Cyt C is released from the mitochondria and forms a complex with Apaf-1 and Caspase-9, after which Caspase-9 activates the downstream protease, Caspase-3, to cleave cell substrates, leading to apoptosis.^23^^,^^24^ Various extracts have been shown to induce apoptosis; for instance, the ethanol extract of *Hizikia fusiforme* induces apoptosis by activating the extrinsic and intrinsic apoptotic pathways and by promoting ROS-dependent inactivation of PI3K/Akt signaling in B16F10 cells.^25^ Meanwhile, isorhamnetin suppresses Akt phosphorylation and NF-κB translocation to induce melanoma B16F10 cell apoptosis.^26^ These findings suggest that the melanin content could be reduced through the apoptotic pathway that it was a beneficial process for reducing melanin synthesis. In this study, QHP did not only reduce melanin content by Akt-signaling pathway in cultured cancer cells and rats but also QHP could accelerate A375 cell apoptosis via three mechanisms, which showed that mitochondrial apoptosis was an active process for tumor cells to reduce melanin, being conducive to the reduction of melanin content.

Given that melanin synthesis begins with the oxidation of l-tyrosine and/or L-DOPA to dopaquinone, which serves as a substrate for eumelanin and pheomelanin synthesis,^1^ oxidative stress is a crucial component of this pathway. Therefore, we analyzed the antioxidant indexes (DPPH scavenging rate, reducing activity, chelation rate) and Tyr activity of QHP. SOD plays a vital role in the balance between oxidation and antioxidation, which is conducive to delayed skin aging, antioxidation, and the removal of colored skin spots.^27^ MDA is an end-product of lipid peroxidation, hence, its content level can indicate the degree of lipid peroxidation in the body.^28^ Therefore, we further measured the effect of QHP intervention on antioxidant indicators (SOD and MDA) of skin in the animal model. Our results showed that QHP protected against UVB-induced skin damage and that the QHP group showed significantly lower SOD and MDA levels than the model group (Figures 6C and 6D). Oxidative stress can reportedly mediate cell apoptosis through the mitochondrial pathway.^29^ QHP has an excellent antioxidant effect and can enhance SOD activity (Figure 2D), which effectively cleared free radicals from the A375 malignant melanoma cell line, reducing TYR activity and melanin content (Figures 2B and 2C). While cancer cells prevent programmed cell death, namely cell apoptosis, QHP has accelerated cell apoptosis through a mitochondria-mediated apoptosis pathway, thus slowing the process of melanin synthesis (Figure 3).

### Limitations of the study

The QHP we obtained did not resolve the specific structure, so we did not know the specific components that played the role of reducing melanin content. The specific binding sites and inhibitory mechanisms of peptides to tyrosinase were also unclear. Although QHP could reduce melanin content through apoptosis, there was still a lack of in-depth research.

## STAR★Methods

### Key resources table

**Table:** 

REAGENT or RESOURCE	SOURCE	IDENTIFIER
**Antibodies**		

Cytochrome C (Cyt C)	Cell Signaling Technology	Cat# 11940T; RRID: AB_1271052
GAPDH	Santa Cruz Biotechnology	Cat# M20006s; RRID: AB_1067058
MITF	Abcam Technology	Cat# TP53061; RRID: AB_317132
Akt	Abcam Technology	Cat# T55561; RRID: AB_398156

**Critical commercial assays**		

FITC Annexin V Apoptosis Detection Kit	Becton Dickinson and Company	Cat# 556570
Mitochondrial Membrane Potential Assay Kit With JC-1	Solarbio Science & Technology Co., Ltd.	Cat# M8650

**Experimental models: Cell lines**		

Human: A375 cells	Dalian Meilun Biotechnology Co., Ltd.	Cat# PWE-HU015

**Other**		

Rats: Female specific pathogen-free (SPF) Sprague Dawley rats	Jinan Pengyue Experimental Animal Breeding Co., Ltd (Jinan, China).	Weifang Medical University Ethics Committee (ID 2021SDL366).

**Oligonucleotides**		

Primers for target genes, see table in qRT-PCR analysis section	This paper	N/A

### Resource availability

#### Lead contact

Further information and requests for resources and reagents should be directed to and will be fulfilled by the lead contact, Fengxiang, Zhang (zfx0515@163.com).

#### Materials availability

This study did not generate new unique reagents.

### Experimental model and subject details

#### Cell lines

A 375 cell (Cat# PWE-HU015) was purchased from Dalian Meilun Biotechnology Co., Ltd. (Dalian, China). A375 cells were cultured in complete medium (DMEM containing 10% FBS and 1% penicillin–streptomycin solution) at 37 °C in a 5% CO_2_ incubator. The above cell lines were used directly from the commercial sources and cultured according to manufacturer suggestions.

#### Other

Female specific pathogen-free (SPF) Sprague-Dawley rats were purchased from Jinan Pengyue Experimental Animal Breeding Co., Ltd (Jinan, China). Rat studies were performed in accordance with the Guide for the Care and Use of Laboratory Animals published by the European Commission and approved by the Weifang Medical University Ethics Committee (ID 2021SDL366). The rats (160–180 g) were acclimatised for 1 week by housing at 25 ± 3°C with a relative humidity of 40% and *ad libitum* access to standard chow and water. UVB injury was then induced to create a model of hyperpigmentation, as described previously with slight modifications.^30^ Briefly, after the rats were anesthetized with 10% chloral hydrate, each rat of the back (5 cm × 5 cm) was shaved and exposed to UVB radiation (280–305 nm) at a dose of 500 mJ/cm^2^ per exposure for 2 weeks.

### Method details

#### Materials and reagents

The crude peptides from quinoa husk (C-QHP) were provided by the Weifang Key Laboratory for Food Nutrition and Safety of Weifang Medical University (Weifang, China). To obtain C-QHP, proteins were extracted from quinoa husk and hydrolyzed into peptides with alkaline protease (1:10, crude protein to water) under a stable pH 8.0 for 3 h A375 cells—a malignant melanoma cell line—Cell Counting Kit-8 (CCK-8), fetal bovine serum (FBS), and Dulbecco’s modified eagle’s medium (DMEM) were acquired from Dalian Meilun Biotechnology Co., Ltd. (Dalian, China). Alkaline protease was acquired from Novozymes (China) Biotechnology Co., Ltd. Monoclonal antibodies against β-catenin and GAPDH were purchased from Santa Cruz Biotechnology (Dallas, Texas, USA). MITF, TYR, TRP-1, TRP-2, Akt, and horseradish peroxidase (HRP)-conjugated secondary antibodies were purchased from Abcam Technology (Cambridge, UK). Cytochrome *c* (Cyt C) and glycogen synthase kinase 3β (GSK3β) were purchased from Cell Signaling Technology (Danvers, MA, USA). Superoxide dismutase (SOD), malondialdehyde (MDA), enhanced chemiluminescence (ECL), polyvinylidene fluoride (PVDF) film, Tyrosinase Activity Detection kit (the article number BC4055) and a Mitochondrial Membrane Potential Assay Kit With JC-1 were obtained from Solarbio Science & Technology Co., Ltd. (Beijing, China). The FITC Annexin V Apoptosis Detection Kit was purchased from Becton, Dickinson, and Company (New Jersey, USA). The RNA Extraction Kit, PrimeScript RT Reagent Kit, and TB Green Premix Ex Taq II were purchased from Takara Biomedical Technology (Beijing) Co., Ltd. Female specific pathogen-free (SPF) Sprague-Dawley rats were purchased from Jinan Pengyue Experimental Animal Breeding Co., Ltd (Jinan, China). All other reagents were of analytical grade.

#### Purifying and screening C-QHP

C-QHP were dissolved in deionised water to a concentration of 10 mg/mL and filtered through a 0.22-μm membrane. The samples (10 mL) were loaded onto a macroporous adsorption resin filtration column (2.6 × 30 cm) after the column was balanced with deionised water. Impurities were washed from the samples with distilled water. When the baseline was stable, the polypeptide was purified by gradient with 30% ethanol or 80% ethanol at a flow rate of 2 mL/min and absorbance detection at 220 nm. Next, the different fractions were collected. Subsequently, the fractions were freeze-dried and the TYR inhibition rate, DPPH scavenging rate,^31^ reducing activity,^32^ and chelation rate^33^ were determined.

The purified peptide was prepared into 1, 5, and 10 mg/mL. According to the dosage in below table, the solution was added into 96 well plates with shaking at 25 °C for 20 min. The absorbance value was then obtained with an enzyme standard instrument (multiskan go, Thermo Fisher) at 475 nm. With ascorbic acid (Vitamin C, Vc) as the positive control, three tests were performed in parallel for each group. The tyrosinase inhibition rate was calculated according to the following formula:

**Table 3:** Reactant addition dose

Reactant	Blank	Blank Control	Sample Blank	Sample
PBS (μL; pH 7.0, 0.02 M)	140	120	100	80
L-Tyrosine (μL; 1.5 × 10^−3^ M)	60	60	60	60
Sample (μL)	–	–	40	40
TYR (μL; 0.33 U/μL)	–	20	–	20

Inhibitionrate(%)=(1−ODsample/ODcontrol)∗100

The molecular weight distributions of the different fractions were determined via high-performance liquid chromatography, using acetonitrile/water/trifluoroacetic acid (45/55/0.1, v/v/v) as mobile phase, TSK-GEL G2000 SWXL chromatographic column (300 mm × 7.8 mm), detection wavelength of 220 nm, flow rate of 0.5 mL/min, and injection volume of 20 μL. Cyt C (12,500 Da) and bacteriostatic peptide (6,500 Da), aprotinin (1,450 Da), glycine-glycine-tyrosine-arginine (Gly-Gly-Tyr-Arg, 451 Da), and glycine-glycine-glycine (Gly-Gly-Gly, 189 Da) were employed as standards. The amino acid compositions were determined using an amino acid analyser.

#### Cell culture and cytotoxicity assay

A375 cells were cultured in complete medium (DMEM containing 10% FBS and 1% penicillin–streptomycin solution) at 37 °C in a 5% CO_2_ incubator. The cells were seeded in 96-well plates (5 × 10^3^ cells/well) and cultured for 24 h. Following cell adhesion to the plate, culture media was removed and discarded. Different concentrations QHP (0.10–0.40 mg/mL) were dissolved in complete medium, and 200 μL were added to each well. The control group comprised 200 μL of complete medium. Cells were cultured for 24, 48, and 72 h; thereafter, the complete media was replaced with CCK-8 culture medium and cultured for an additional 4 h at 37 °C. Cell survival rate was calculated by measuring absorbance at 450 nm.

#### Determining TYR activities and melanin content in cells

Cells were seeded in 6-well plates (5 × 10^4^ cells/well) for 24 h and subsequently incubated with various concentrations of QHP for 48 h (0.1–0.35 mg/mL). Parallelly, the control group was incubated with DMEM lacking QHP. The medium was discarded, and cells were rinsed twice with PBS before the addition of 1 mL of 5% Triton X-100 to each well. Cells were then further lysed by performing multiple freeze–thaw cycles (3-5 times). TYR was released into the supernatant, which was collected after centrifugation at 10,000 rpm for 20 min. Next, 100 μL of supernatant and 100 μL of 0.1% l-dihydroxyphenylalanine (l-DOPA) solution were added into wells of a new 96-well plate. The plates were incubated at 37 °C for 2 h, after which the absorbance at 475 nm was measured and TYR activity was calculated according to the kit instructions. The TYR activity of the control group was defined as 100%.

The cell culture process has been described previously. The cells were then digested with 500 μL of trypsin after discarding the culture medium, flushed with cold PBS, collected and rinsed again with PBS. Subsequently, NaOH solution (1 mL, 1 mol/L) was added to the collected cells and incubated at 80 °C for 2 h; 100 μL of each concentration was then added to a new 96-well plate to measure the absorbance at 475 nm. Melanin content was calculated according to the following formula:

Melanincontent(%)=OD(samplegroup−sampleblankgroup)/ODcontrolgroup∗100

The group containing only medium served as the control group.

#### Apoptosis assay

Cells were seeded in 6-well plates (5 × 10^4^ cells/well) for 24 h and subsequently cultured in presence of various QHP concentrations (0.1–0.35 mg/mL) for 48 h. The control group was incubated with the same volume of DMEM without QHP. Culture media was removed, cells were digested with trypsin, without EDTA, and washed with PBS. The cells were transferred to a new centrifuge tube, centrifuged at 1000 ✕ g for 5 min, and the supernatant was discarded. Cells were resuspended in PBS and counted. Next, 1–5 ✕ 10^5^ cells were centrifuged at 1000 ✕ g for 5 min, the supernatants were discarded, and binding solution (500 μL) was gently added. Annexin V-FITC (5 μL) was then added and mixed gently followed by addition of 5 μL of propidium iodide and gentle mixing. The cells were incubated for 10 min at room temperature (20–25 °C) in the dark, and then analyzed by flow cytometry. Alternatively, 1 mL of JC-1 staining solution was added to cells, evenly mixed, and incubated at 37 °C for 20 min. The supernatant was discarded, and cells were washed twice with JC-1 staining buffer. Subsequently, 2 mL of cell culture medium was added and observed under a fluorescence microscope (Olympus IX 73, Japan). DNA damage was assessed by comet assays with a DNA Damage Kit, and the data were analyzed using cometA software.

#### Transcriptome sequencing and bioinformatics analysis

A375 cells were seeded in cell culture flasks (5 × 10^6^ cells/well) and incubated for 24 h, after which they were exposed to 0.30 mg/mL of QHP for 48 h. After discarding the culture medium and flushing the cells with cold PBS, the cells were digested with trypsin. The trypsin digestions were terminated via addition of medium. The cells were then snap-frozen in liquid nitrogen and stored at −80 °C. Transcriptomes were sequenced by Suzhou Panomek Biomedical Technology Co., Ltd.

#### Western blot analysis

Western blot analysis was performed to analyze the cellular expression levels of Akt, GSK-3β, β-catenin, MITF, TYR, TRP1, TRP2, and Cyt C. Equal amounts of protein were obtained from cells; however, it should be noted that the proteins used to determine Cyt C were extracted from the mitochondria by the Mitochondria Isolation Kit. Protein concentrations were determined using the bicinchoninic acid assay method. Then, protein samples were separated by sodium dodecyl sulphate-polyacrylamide gel electrophoresis and transferred to PVDF membranes. Each membrane was blocked in 5% skim milk for 2 h after rinsing three times with Tris-buffered saline with Tween 20 (TBST; 5 min/rinse step). Each membrane was then incubated with an appropriate primary antibody (1:1500) at 4 °C for 1 h. After removing unbound primary a via rinsing with TBST thrice (5 min/rinse step), the membranes were incubated with HRP-conjugated secondary antibodies for 2 h at room temperature (25 °C). Finally, the signals were visualised using ECL detection reagents and quantified by densitometry using ImageJ software. The experiments were repeated three times.

#### qRT-PCR analysis

To determine the relative gene expression levels of Akt, Gsk3β, β-catenin, MiTF, TYR, TRP1, and TRP2, total RNA was extracted from cells using a Universal RNA Extraction Kit (TaKaRa). The RNA samples were quantified and converted into cDNA using the PrimeScript RT Reagent Kit (TaKaRa, Japan). The sequences of the primer pairs used for the qRT-PCR analysis are shown in below table. For the real-time PCR step, 2 μL of cDNA was amplified using TB Green Premix Ex Taq II (TaKaRa). The PCR reaction comprised a denaturation step at 95 °C for 30 s, followed by 40 cycles of denaturation at 95 °C for 5 s, and annealing at 60 °C for 30 s. The dissolve program comprised: 95°C for 15 s, 60°C for 60 s, and at 95°C for 1 s. Each sample was analyzed in triplicate. Relative gene-expression levels were calculated using the 2−ΔΔCt method.

**Table 4:** Sequences of primers for target genes of the amplified fragment

Gene	Primer sequences
Akt	F: 5′-GCCCTCAAGTACTCATTCCAG-3′
	R: 5′-ACACAATCTCCGCACCATAG-3′
Gsk-3β	F: 5′-CACCTGCACTCTTCAACTTTAC-3′
	R: 5′-CACGGTCTCCAGCATTAGTATC-3′
β-catenin	F: 5′-TGCAGTTCGCCTTCACTATG-3′
	R: 5′-ACTAGTCGTGGAATGGCACC-3′
MITF	F: 5′-AGGACCTTGAAAACCGACAG-3′
	R: 5′-GGTGGATGGGATAAGGGAAAG-3′
TYR	F: 5′-CCCAGAAGCCAATGCACCTA-3′
	R: 5′-ATAACAGCTCCCACCAGTGC-3′
TRP1	F: 5′-TTCATTGGCACCTGCTTTGC-3′
	R: 5′-TCACAGCTCCAACGAAGGAC-3′
TRP2	F: 5′-CCTACCGCCTTCGAGTCATC-3′
	R: 5′-TCCCAGGCATAGTCAGCTCT-3′
GAPDH	F: 5′-TGAACGGGAAGCTCACTGG-3′
	R: 5′-GCTTCACCACCTTCTTGATGTC-3′

#### Animal experiment

The rats were assigned to the following four groups (n = 6/group): (i) control group, not subjected to UVB treatment; (ii) model group, subjected to UVB-induced injury for 2 weeks and allowed to recover naturally for 4 weeks; (iii) positive-control group, subjected to UVB-induced injury for 2 weeks and treated for 4 weeks with 5 mg/mL Vc, dissolved in ethanol: propylene glycol (3:7, V/V); and (iv) QHP group, subjected to UVB-induced injury for 2 weeks and treated for 4 weeks with 5 mg/mL QHP, dissolved in ethanol: propylene glycol (3:7, V/V).

Skin-biopsy specimens were fixed in 3% paraformaldehyde solution for 24 h. The slices were prepared by Jinan Saier Biological Technology Co., Ltd. SOD and MDA levels were measured using the corresponding kits. After the rats were anesthetized, skin samples were collected from rats for western blot and qRT-PCR assays, as described in "Western blot analysis" and "qRT-PCR analysis".

### Quantification and statistical analysis

Statistical analyses were performed using GraphPad Prism (version 8.0.2) and Origin (version 9.0) software. The results were reported as mean ± SD and analyzed by ANOVA-multiple comparisons. p-values <0.05 or <0.01 were considered statistically significant.

## Data Availability

Data reported in this paper will be shared by the lead contact upon request. This paper does not report original code. Any additional information required to reanalyze the data reported in this paper is available from the lead contact upon request.
